# Functional Connectivity in the Default Mode Network During Rumination in Depression: A Systematic Review

**DOI:** 10.1192/bjo.2025.10214

**Published:** 2025-06-20

**Authors:** Sahar Riaz, Orla Mitchell, Darren Roddy, Michael Connaughton

**Affiliations:** RCSI, Dublin, Ireland

## Abstract

**Aims:** The Default Mode Network (DMN) is a network of brain regions that are functionally connected and become active during self-directed thought, introspection, and rumination. Rumination refers to the repetitive and passive focus on distressing thoughts, often linked to negative emotional states. Specifically, an increase in the brooding type of rumination is associated with severity of depression. Functional magnetic resonance imaging (fMRI) research offers a unique perspective on how the functional connectivity of the DMN is involved in rumination, shedding light on its neurobiological underpinnings. This systematic review aims to synthesise existing literature that explored the functional connectivity of the DMN in individuals with depressive disorders during episodes of rumination.

**Methods:** This systematic review investigated activity in DMN in patients with depression using resting state fMRI scans. Literature search was done on PubMed, Medline and Cochrane using search terms “depression OR Major Depressive Disorder OR depressive episode” AND “ruminat*” AND “functional MRI OR fMRI”. 324 studies were identified from the three databases, and after removing 42 duplicates, 274 studies were selected for title and abstract screening.

Abstracts were assessed for eligibility using the following inclusion criteria: original studies in peer-reviewed journals, clinical diagnosis of depressive disorder, measurement of rumination using a validated scale and resting state or task-based fMRI. 193 studies were excluded, and 58 studies were moved to full-text review. Intervention studies were also excluded at this stage. Following the above criteria, 25 studies were selected for full-text review.

**Results:** Out of the 25 studies, 9 used task-based fMRI and 16 used resting state fMRI. Only resting state fMRI studies were included for data extraction. Results from the 16 studies showed that depressed people had both increased and decreased functional connectivity between different regions of the brain during brooding rumination. The connectivity within the DMN was increased, while connectivity between DMN and other areas of brain, including between DMN and TPN (task-positive network) was reduced, when compared with healthy controls.

**Conclusion:** This review shows widespread associations between depression, rumination and functional connectivity within and between various brain regions. Increase of functional connectivity within the DMN during depression might be responsible for the increase in brooding rumination seen in depressed individuals. A decrease in connectivity of DMN to other areas of the brain might result in difficulties for depressed individuals to switch from a ruminating state into the executive network mode. Overall, this review provides an overview of the neurobiological underpinnings for the increase in brooding rumination in depression.

